# New Appliances and Things Medical

**Published:** 1908-06-20

**Authors:** 


### NEW APPLIANCES & THINGS MEDICAL.

### CYLLIN OBSTETRICAL LUBRICANT.

The various preparations of Cyllin have already established themselves in the eyes of the medical profession as efficient disinfectants for a large number of purposes. The manufacturers, Jeyes' Sanitary Compounds Company, Limited, of 64 Cannon Street, London, E.C., have now brought out a new preparation which should increase the value of Cyllin in obstetrical work. We have lately received from them a sample collapsible tube of Cyllin Obstetrical Lubricant. This substance is a clear pale-brown jelly free from grease and readily soluble in water. It is therefore easily removed from the hands after it has served its purpose. We have found it singularly free from irritant and caustic effects upon mucous membranes or toxic after-effects, and the Cyllin which enters into its composition appears to be present in an active form. This lubricant can therefore be recommended to medical men as a safe and reliable substitute for carbolised vaseline, glycerine, etc., in obstetrical and gynæcological examinations. An effective antiseptic lubricant such as this, without toxic or greasy properties, is a useful addition to the midwifery equipment.

### WHITEWAY'S PURE DEVONSHIRE CIDER.

(22 Albert Embankment, London, S.W.).

We have received several bottles of this cider, and have examined them carefully. One of them is worthy of notice, being non-alcoholic. To this the term cydrax has been given. It is free from alcohol, has a good taste, is not too sweet, and, further, does not possess a high acidity. The remaining ciders contain about the average amount of alcohol, which is, of course, very low, and vary chiefly *inter se* with regard to dryness.

We think a more general consumption of cider would be in the interests of the community at large. It is a very light alcoholic beverage, and contains natural salts, which experience has shown to be beneficial to the human organism. Its acidity is mostly due to the presence of malic acid, and hence is of quite a different type to the acidity of many wines. Whiteway's ciders are of good quality and taste, and bear indubitable evidence of the careful and up-to-date manner in which they are prepared.

